# Considerations for using ETEC and *Shigella* disease burden estimates to guide vaccine development strategy

**DOI:** 10.1016/j.vaccine.2017.09.083

**Published:** 2019-11-28

**Authors:** Divya Hosangadi, Peter G. Smith, Birgitte K. Giersing

**Affiliations:** a Johns Hopkins Bloomberg School of Public Health, Baltimore, MD 21205, USA; b MRC Tropical Epidemiology Group, London School of Hygiene & Tropical Medicine, London, UK; c Initiative for Vaccine Research, World Health Organization, CH-1211 Geneva 27, Switzerland

## Abstract

Enterotoxigenic *E. coli* (ETEC) and *Shigella* are enteropathogens causing significant global morbidity and mortality, particularly in low-income countries. No licensed vaccine exists for either pathogen, but candidates are in development, with the most advanced candidates potentially approaching pivotal efficacy testing within the next few years.

A positive policy recommendation for introduction of any vaccine, following licensure, depends on evidence of vaccine cost-effectiveness and impact on morbidity and mortality. The mortality estimates for these two pathogens have fluctuated over recent years, which has led to uncertainty in the assessment of their relative public health importance for use in low and middle-income countries.

This paper summarizes the various ETEC and *Shigella* disease burden estimates, based on a review of current literature and informal consultations with leading stakeholders in enteric disease modelling. We discuss the factors that underpin the variability, including differences in the modelling methodology; diagnostic tools used to ascertain diarrheal etiology; epidemiological setting; the data that are available to incorporate; and absolute changes in the total number of diarrheal deaths over time. We consider the further work that will strengthen the evidence needed to support future decision making with respect to recommendations on the relative utility of these vaccines.

## 1 Introduction

Recent global estimates attribute more than a million deaths per year to diarrhea across all age groups and approximately 4% of total global disability-adjusted life years (DALYS) [1], [2], [3]. Despite reductions in mortality, diarrheal morbidity remains high, and the condition remains a major burden in low and middle income countries (LMICs) in Africa and Asia, where water quality and sanitation are poor [4], [5], [6], [7]. *Shigella* and ETEC are major causes of diarrhea in infants and school-age children, as well as in adults and travelers to endemic regions [8], [9], [10], [11], [12], [13]. However, global mortality estimates for these pathogens have fluctuated since 2010, and estimated mortality rates are now lower than ever previously reported. There is some debate as to whether development of stand-alone vaccines against these pathogens is warranted, or whether combination vaccine approaches, or other interventions, should be prioritized.

Both ETEC and *Shigella* are spread via fecal-oral transmission, through contaminated food or water, and human-to-human contact [14], [15]. Household contacts of shigellosis cases may be particularly susceptible to infection, with possible transmission by houseflies [16], [17]. ETEC strains (producing heat-labile toxin [LT] and/or heat-stable type toxin [ST]) produce fimbrial or non-fimbrial adhesins that enable attachment to host epithelial cells, allowing them to colonize the small intestine. They then release LT and/or ST enterotoxins that disrupt electrolyte homeostasis, leading to fluid loss and eventually secretory (watery) diarrhea [18]. Both the human (STh) and porcine (STp) genotypes of ST are associated with human disease [19]. Given the mechanism of ETEC pathogenesis, development of vaccine candidates has focused on presentation of appropriate colonization factor antigens (CFA) adhesins and enterotoxins. However, at least 25 immunologically distinct CFAs have been identified and it remains challenging to overcome colonization factor and toxin heterogeneity [20], [21].

*Shigella* spp. are invasive organisms causing both watery diarrhea and dysentery following internalization into specialized endothelial (M) cells and activation of a cascade of immunological responses that cause inflammation and ultimately destruction of the bowel [22], [23]. Watery diarrhea typically precedes dysentery and results from the action of enterotoxins in the jejunum, whereas bloody diarrhea results from invasion of the colonic epithelium. To date, four species and 49 serotypes have been identified that include *S. dysenteriae* (15 serotypes)*, S. flexneri* (13 serotypes), *S. boydii* (20 serotypes) and *S. sonnei* (1 serotype). The major focus of vaccine development effort is directed to *S. flexneri* which accounts for 60% of endemic disease in infants and children in LMICs, whereas *S. sonnei* causes the majority of cases in industrialized countries and is an important cause of travelers’ diarrhea. Serotypes are defined based on the carbohydrate composition of the surface O-antigen, and vaccine development approaches are broadly based on serotype-specific targeting of these antigens, or conserved antigens, such as invasion plasmid antigen (Ipa) proteins [24].

Whole cell and subunit ETEC and *Shigella* vaccine candidates are currently in clinical trials [22], [24], [25]. Distribution of strains, antigenic components, and disease severity differ between developed countries and LMICs and can vary by age group [26], [27]. The most common diarrheagenic ETEC colonization factors include CFA/I, CS3 and CS6, while the most common *Shigella* serotypes are S. *flexneri* 2a, 6, 3a, and S. *sonnei* [28], [29]. The most advanced ETEC and *Shigella* vaccine candidates include these components in the form of killed whole cells [24], [25], [28] that may be suitable for combination [30].

Several studies have indicated repeated enteric infections can be associated with long term sequelae and diseases, including malnutrition, stunting, pneumonia and arthritis, in both LMICs and developed countries [4], [31], [32], [33]. The effects of these infections are most profound in infants and school-age children, and can hinder cognitive development, school performance, and economic stability [34], [35], [36]. In addition, the rise of antibiotic resistance, and a recent safety warning related to treatment with the antibiotic Ciprofloxacin, further underscore the persisting unmet need for effective vaccines against these pathogens [37], [38].

Although ETEC and *Shigella* vaccine candidates are at least five to 10 years from licensure, conclusions regarding the public health need for vaccines based on currently available burden estimates inform vaccine investment decision-making today, particularly with respect to a potential combination vaccine [30]. Such decisions impact critical components of vaccine development strategy, including clinical and regulatory pathways, formulation, and presentation optimization. In addition, accurate disease burden estimates provide baseline data to assess future vaccine impact, and help identify clinical trial sites for rapid and appropriately powered vaccine efficacy assessments. It is important, therefore, to understand the underlying causes for the uncertainties and possible limitations of *Shigella* and ETEC disease burden estimates.

## 2 Current disease burden estimates

Our discussion focuses on published ETEC and *Shigella* disease burden estimates that are generally based either on secondary, extrapolated information taken from systematic reviews, or primary data directly gathered at community or clinic levels. The Global Burden of Disease (GBD) Mortality and Causes of Death Collaborators, Child Health Epidemiology Reference Group/Maternal Child Epidemiology Estimation (CHERG/MCEE), the Foodborne Disease Burden Epidemiology Reference Group (FERG), and other secondary analyses have based their conclusions on a combination of systematic literature reviews, government vital records and verbal autopsies, and modelling [1], [2], [10], [14], [39], [40].

Estimates of mortality attributable to ETEC and *Shigella* depend on the data sources used, geographical range, year of reporting, case definitions, age groups, and methodology. For example, successive iterations of the GBD estimates, from 2010 to 2015, used modified methodology and new data inputs [1], [2], [39], [40]. Consequently, burden estimates vary and cannot be easily compared.

### 2.1 Estimates derived from systematic reviews and models

Table 1 shows recent estimates of ETEC and *Shigella* deaths in children under five years. The most recently published estimates from GBD are for the year 2015 [1], [40], and from CHERG/MCEE for the year 2011 [10]. In children under five years, the GBD 2015 analysis estimated *Shigella* deaths to be 54,905 (27,026–94,731), in contrast to the CHERG/MCEE 2011 analysis of 28,000 (12,000–53,000). The under -five-years mortality estimate due to ETEC was 23,649 (9,553–44,337) according to GBD in 2015, and 42,000 (20,000–76,000) according to CHERG/MCEE in 2011 [1], [10], [40]. Of note, estimated *Shigella* deaths in the GBD analyses for the years 2013 and 2015, increased from 33,400 (24,900–43,500) to 54,905 (27,026–94,731), respectively, whereas the estimated number of ETEC deaths between the years remained similar [1], [39].

**Table 1:** Global Estimates of Deaths Among Children Under Five.

Study	FERG [14]^a^	CHERG/MCEE [10]	GBD 2013 [39]	GBD 2015 [1], [40]
Year of publication	2015	2013	2015	2016
Time period of data collection	1990–2012	1990–2011	1990–2013	1980–2015
Year represented in data	2010	2011	2013	2015
Geographical range of estimates	194 countries	Global	188 countries	195 countries and territories
Number of pathogens assessed	22	13	13	13
All-cause diarrheal deaths (uncertainty range)	91,621 (62,442–132,707)	712,000 (491,000–1,049,000)	474,900 (398,100–545,000)	499,000 (447,000–558,000)
ETEC deaths (uncertainty range)	14,056 (7,045–26,784)	42,000 (20,000–76,000)	23,100 (17,000–30,400)	23,649 (9,553–44,337)
*Shigella* deaths (uncertainty range)	8,863 (3,250–20,925)	28,000 (12,000–53,000)	33,400 (24,900–43,500)	54,905 (27,026–94,731)

a Estimates are of deaths attributable specifically to **foodborne transmission** of these two pathogens.

With respect to deaths at all-ages, the GBD estimate of annual *Shigella*-attributable deaths decreased from 122,800 (97,400–149,600) in the year 2010 to 73,900 (58,900–93,800) in 2013, and subsequently increased to 164,300 (85,000–278,700) in 2015 [1], [2], [39]. Likewise, the ETEC mortality estimates have fluctuated from 120,800 (95,700–147,600) in 2010 to 59,200 (44,200–77,700) in 2013, followed by a comparatively more modest increase to 74,100 (29,900–137,900) in 2015 (Table 2) [1], [2], [39]. However, the uncertainty intervals on these estimates are wide and overlapping.

**Table 2:** Global Estimates of Deaths Among All Age Groups Combined.

Study	FERG [14]	GBD 2010 [2]	GBD 2013 [39]	GBD 2015 [1]
Year of publication	2015	2012	2015	2016
Time period of data collection	1990–2012	1980–2010	1990–2013	1980–2015
Year represented in data	2010	2010	2013	2015
Geographical range of data	194 countries	187 countries	188 countries	195 countries and territories
Number of diarrhea causing pathogens assessed	22	10	13	13
All-cause diarrheal deaths (uncertainty range)	1,092,548 (892,999–1,374,238) a350,686 (240,030–524,042)	1,445,800 (1,278,900–1,607,000)	1,264,100 (1,151,200–1,383,200)	1,312,100 (1,233,600–1,391,300)
Number of ETEC deaths (uncertainty range)	73,857 (53,851–103,026) a26,170 (14,887–43,523)	120,800 (95,700–147,600)	59,200 (44,200–77,700)	74,100 (29,900–137,900)
Number of *Shigella* deaths (uncertainty range)	65,796 (46,317–97,036) a15,156 (6,839–30,072)	122,800 (97,400–149,600)	73,900 (58,900–93,800)	164,300 (85,000–278,700)

a Estimates correspond to deaths specifically attributed to **foodborne transmission** of the pathogen.

Contrary to GBD estimates, but in line with estimates published by CHERG/MCEE, FERG estimates for overall ETEC mortality are higher at all ages than for *Shigella*, at 73,857 (53,851–103,026) vs 65,796 (46,317–97,036), respectively [14]. Furthermore, the number of ETEC deaths specifically attributable to foodborne transmission are determined to be higher than that of *Shigella* at all ages (Table 2: 26,170 (14,887–43,523) vs 15,156 (6,839–30,072)*,* respectively) with approximately half the burden occurring in the under -five-year age group [14]. Both pathogens had a greater number of illnesses among those over five years, but a greater number of deaths among those under five. This was particularly true for *Shigella*, which had more than twice the number of illnesses among those over five compared those under five [14].

Year lost due to disability (YLD) and disability adjusted life-years (DALY) data further demonstrate the burden of these two pathogens. Colombara et al. concluded that both ETEC and *Shigella* were among the top five enteric pathogens causing diarrhea-associated YLDs [41]. GBD 2010 data indicate that a slightly greater number of YLDs are attributable to ETEC than *Shigella* (15 per 100,000 vs 11 per 100,000), and that DALYS attributable to each pathogen are relatively similar to each other (6,894,000 for ETEC vs. 7,052,000 for *Shigella*). However, uncertainty intervals corresponding to each pathogen overlap for both DALYS and YLDs [3], [42]. FERG attributed a greater number of DALYs to ETEC (30 (17–51) per 100,000) than *Shigella* (18 (8−3 7) per 100,000). Table 3 shows additional DALY and YLD data [14].

**Table 3:** Reported YLD and DALY estimates.

Study	Year reported in data	Age group	Total ETEC YLDs [42]	Total *Shigella* YLDs [42]	Total ETEC DALYS [3]	Total *Shigella* DALYS [3]
GBD 2010 (uncertainty range)	2010	All ages	1,065,000 (649,000–1,643,000)	744,000 (440,000–1,147,000)	6,894,000 (5,619,000–8,286,000)	7,052,000(5,676,000–8,466,000)
FERG	2010	All ages	–	–	5,887,541 (4,190,610–8,407,186) [14]	5,407,736 (3,771,300–8,107,456) [14]

A systematic review by Fischer-Walker et al. in 2010 found that ETEC was about twice as common as *Shigella* among inpatient cases of diarrhea (14% vs 6.5% of cases, respectively) across all age groups, while *Shigella* was about three times more common than ETEC in outpatient settings (19.6% vs 5.9%, respectively) [43]. Among older children and adults, Lamberti et al. concluded that the burden of morbidity and mortality associated with ETEC and *Shigella* among those over five years old in Africa and South Asia was substantial (19,451 deaths due to *Shigella* spp. and 42,973 due to ETEC in AFR, and 20,691 due to S*higella* spp. and 45,713 due to ETEC in SEAR in 2010) [11]. An improved understanding of the proportional distribution of inpatient vs outpatient cases of *Shigella* and ETEC is needed to better inform burden assessments, and to rationalize the most cost-effective vaccination strategy.

To summarize, there has been significant deviation in various iterations of the mortality estimates produced by the same groups, as well as variances in the absolute estimates reported between different groups. It is unclear which estimates are the most accurate. This lack of clarity risks product development decision making, and may ultimately jeopardize policy review for vaccine introduction, since these are driven by burden of disease data.

### 2.2 Estimates derived from specific epidemiological studies

The Global Enteric Multi-center Study (GEMS) and the Etiology, Risk Factors, and Interactions of Enteric Infections and Malnutrition and the Consequences for Child Health and Development (MAL-ED) are epidemiologic studies that gathered primary data directly from high burden, low- income communities [9], [12], [44]. GEMS was a clinic-based, three-year, case-control study conducted at seven sites in Africa and Asia that investigated moderate-to-severe diarrhea (MSD) in children under five years [9]. Children were also visited 2–3 months after enrolment. Three age strata were evaluated: 0–11 months, 12–23 months and 24 – 59 months.

MAL-ED was a longitudinal community-based birth cohort study conducted in 8 sites in Africa, Latin America and Asia that analyzed pathogen-specific diarrheal burden in children between 0–11 months and 12–24 months. The differences in structure and design of these two studies are important to consider when assessing their results (Section 4), and have been discussed in a recent review [45].

GEMS, which was conducted at sites that had not introduced rotavirus vaccine, concluded that most attributable cases of moderate-to-severe diarrhea were due to rotavirus, Cryptosporidium, ST-producing ETEC (with or without LT), and *Shigella*, across all study sites and in one or more age strata. The adjusted attributable fraction (AF) of *Shigella* increased as age increased at every site. ST-producing ETEC was a significant pathogen at every site in at least one age stratum and in all age strata at four sites. It was one of the pathogens most associated with risk of death in the  two months following diagnosis (Hazard Ratio 2.0, p = 0.03), however there may be other factors that contributed to, or caused, death between the initial diagnosis and the follow up visit 2 months later [9]. Conversely, ETEC producing LT alone was not a significant cause of moderate-to-severe diarrhea at any site or in age stratum. Of note, the median age of MSD cases with an ST-ETEC infection was 14 months of age, whereas it was 6 months later for *Shigella* at 20 months of age [46]. These findings may have implications for the optimal immunization schedule of these vaccines, and their fit within the Expanded Programme of Immunization (EPI).

In line with the GEMS conclusions, MAL-ED found ST-producing ETEC was more common than *Shigella* in the first year of life [12]. Higher proportions of ST-producing ETEC, compared to LT-producing ETEC, were observed among diarrheal episodes in both the first (1.9% vs 1.3%) and second (3.9% vs 1.2%) years of life, with non-overlapping confidence intervals for ages 12–24 months. It also reported that LT-producing ETEC, ST-producing ETEC and *Shigella* were associated with persistent diarrhea [12]. MAL-ED attributable fractions (AFs) for ST-producing ETEC among children 12–24 months with severe disease were more similar to those reported in GEMS.

Both GEMS and MAL-ED have recently published updated analyses using more sensitive quantitative molecular diagnostic assays [47], [48]. A recent reanalysis of GEMS samples, which included STp, found that the AFs increased by about twofold for *Shigella* and 1.5-fold for ST-ETEC, with *Shigella* showing the largest increase in the second year of life [48]. However, between 2 and 3% of control samples with both watery diarrhea and dysentery were infected with *Shigella* in children over 11 months[46]. The change in the assigned etiology following the GEMS reanalysis highlights the impact on disease burden estimates of utilizing more sensitive and specific analytical methodology for diagnosis. This is further demonstrated by the MAL-ED study involving a subset of specimens that were assessed using qPCR against 19 enteropathogens. With qualitative PCR detection, no pathogens were significantly associated with diarrhea, however quantitative analysis using qPCR revealed that *Shigella*/enteroinvasive *Escherichia coli* infections (OR = 1.47, P = 0.04) were significantly associated with symptomatic diarrhea [47].

Asymptomatic carriage among children without diarrhea was found in both studies and has been observed with both conventional and molecular diagnostic methods, particularly for LT-producing ETEC in MAL-ED. For ST-producing ETEC, prevalence was about twice as high in symptomatic cases compared to non-diarrheal samples in each age cohort of MAL-ED. Interestingly, while the *Shigella* prevalence was notably higher among diarrheal samples compared with the non-diarrheal samples between 12 and 24 months of age in MAL-ED, the difference in prevalence narrowed for the 12–23 month age cohort in GEMS (OR of 2.0 compared to OR = 7.5 for the 0–11 month cohort) [46]. This indicates that levels of asymptomatic carriage can fluctuate across age groups and study settings, and is particularly confounding in LMIC contexts, where children are often infected with multiple enteric pathogens. Studies are ongoing to compare detected pathogen loads, in terms of qPCR threshold cycle (*Cq*) values, and to stratify these with respect to symptomatic and asymptomatic infections. This approach may help to provide greater etiologic accuracy, and is likely to result in further shifts in future disease burden estimates.

These two studies exemplify how severe and less severe diarrheal diseases may have different distributions of etiologies, and how case definition is critical to capture asymptomatic and mild cases of disease. As discussed in Section 5, mild disease is also of significant public health concern.

## 3 Variability in methodology across modelled estimates

### 3.1 Variation among the 2010, 2013 and 2015 GBD estimates

Some aspects of calculating etiologic-specific diarrheal estimates for GBD have changed with each iteration of the estimates [1], [2], [39]. The GBD 2010 pathogen-specific estimates for diarrheal mortality used the International Classification of Disease approach for establishing a single underlying pathogen as a cause of diarrheal death [1], [39]. GBD 2013 and GBD 2015, instead, used a counterfactual approach that considered the prevalence and risk of disease for each diarrheal pathogen, similar to how risk factors were calculated, and used GEMS-derived odds ratios to determine the pathogen-specific attributable fractions [1], [39]. GBD 2013 used data from the original GEMS study. For countries not included in the GEMS study, the mean odds ratios from nearby GEMS sites were used, or an average of odds ratios from all sites were used to determine etiology [39].

The GBD 2015 iteration incorporated data produced from the more sensitive qPCR reanalysis of GEMS samples leading to higher AFs for many pathogens compared to those in GBD 2013 [1], [39], [48]. GBD 2015 estimates incorporated the mean odds ratios of all GEMS sites. Because most diarrheal etiology data used in other studies included in GBD 2015 relied on less sensitive conventional methods, GBD 2015 calculations used a correction factor to adjust prevalence estimates such that they became more comparable to odds ratios from the GEMS reanalysis [1].

While the GEMS data affected global diarrheal burden estimates in GBDs 2013 and 2015, not all trends observed in GEMS were reflected in GBD estimates. For example, while the GEMS reanalysis generally showed an increase in the ETEC AF, a comparable increase in AF did not occur from GBD 2013 to GBD 2015 (4.7–5.6% among all ages, respectively) [1], [9], [39], [48]. Furthermore, the AF for non-typhoidal *Salmonella* is higher than that of ETEC in GBD 2015 (7.7% vs 4.7% children under five years, respectively), but the AF from *Salmonella* spp. was lower than that of ETEC across all age groups in GEMS studies [1], [39]. Fully capturing the complexities of developing recent GBD estimates goes beyond the scope of our report, but could be one of the goals of future consultations or working group discussions aimed at better understanding *Shigella* and ETEC disease burden.

### 3.2 Variation among GBD and other modelled disease burden estimates

Although structural differences between the GBD and CHERG/MCEE models exist, data inputs likely contributed most to the variability between estimates [49]. While GBD used a mix of observational data from inpatient, outpatient, community-based and case-control studies from data sources spanning several decades, CHERG/MCEE used inpatient data from 1990 to 2011 [10]. Approaches used to account for co-infections also differed between the two studies. Importantly, the most currently published pathogen-specific diarrheal CHERG/MCEE estimates do not incorporate the GEMS derived odds ratios to determine pathogen specific AFs because GEMS did not distinguish between inpatient and outpatient cases [1], [10], [49]. Further detail regarding the intricacies of methodologies used by these studies is described in multiple sources [2], [10], [39], [49], [50].

FERG based etiologic distribution of food-borne illness on inpatient, outpatient and community research studies [14]. Like CHERG/MCEE, results from systematic literature reviews were used to estimate attributable fractions. FERG estimated the etiological proportions associated with non-fatal illness for children under five years based on the distribution of pathogens found in the outpatient and community studies, whereas the distribution of pathogens in inpatient settings was assumed to reflect the pathogen prevalence among diarrheal deaths [14], [51]. For people over five years, inpatient studies in addition to outpatient or community studies were used to calculate pathogen prevalence among cases, due to a lack of outpatient or community data in older populations. Pathogens that were not commonly transmitted through food, such as rotavirus, were aggregated as ‘other pathogens’, hence rotavirus was not identified as a top cause of diarrhea in this study [51]. Similarly, Lamberti et al. used the distribution of pathogen etiology among hospitalized cases to determine the etiologic proportions associated with pathogen-specific diarrheal mortality [11], [14]. Because ETEC may be more likely to be found in hospitalized patients than *Shigella*, the focus of these studies on using inpatient data to estimate mortality could possibly underestimate *Shigella* mortality [43]. Also, the incidence of *Shigella* is possibly cyclical over the course of multiple years, contributing to a possible waxing and waning of shigellosis hospitalization rates [52], [53]. Lamberti et al. exclusively assessed *Shigella* and ETEC, rather than all common diarrheal pathogens, which could contribute to potentially higher estimates [11].

## 4 Factors that contribute to variation in disease burden and attribution of etiology for both modelled and epidemiologic estimates

The differences reported across the disease burden estimates discussed, particularly in epidemiologic studies such as GEMS and MAL-ED, are attributable to epidemiological setting, methodological differences in the detection and estimation methods, the data available, as well as to changes in the total number of diarrheal deaths. Factors that influence the estimate outcome are discussed below.

### 4.1 Geographical variation

The studies and systematic reviews discussed above have analyzed data derived from different geographical and socio-economic settings. GBD, FERG, and MCEE/CHERG analyses included data from both low and high income countries [1], [10], [39]. A limitation of all the systematic reviews is that case detection and surveillance are lacking from many countries so that data from surrounding or similar countries must be extrapolated.

Both GEMS and MAL-ED collected primary data from South Asian and Sub-Saharan African LMICs, while MAL-ED also included Latin American sites [9], [12]. In general, the GEMS sites were in more resource-limited areas than the MALED sites, which may have increased exposure and susceptibility to other risks. In GEMS, it was noted that the burden of *Shigella* at the site in Mirzapur, Bangladesh was found to be particularly high, and approximately four times that of the GEMS region with the next highest AF among children in the second year of life (52.2% in Mirzapur vs 12.8% in Basse, Gambia 12–23 mos.) [9]. The GEMS qPCR reanalysis confirmed this trend, and the AFs associated with most pathogens, particularly *Shigella*, increased across almost all sites [48]. Interestingly, the MAL-ED Dhaka site approximately 1000 km away from Mirzapur had one of the lowest *Shigella* AFs among all the study locations, suggesting substantial localized variation in incidence [12]. Higher *Shigella* and ETEC incidence has been reported in the EMR and AFR regions, respectively, underlying likely geographic heterogeneity in burden [11], [51], [54]. Since these data may not be broadly representative of the surrounding region, their inclusion may introduce bias into the estimates, particularly if they are used for extrapolation.

### 4.2 Case definition

There were differences in case ascertainment methods in the GEMS and MAL-ED studies. The clinic-based GEMS study did not distinguish inpatient and outpatient cases, [9], [10] and its case definition of MSD was designed to select for diarrheal disease that may have been fatal in children under five years if left untreated in the community. MAL-ED included both mild diarrhea and MSD cases [12], [55]. MAL-ED used a modified Vesikari score to assess which pathogens were associated with particular symptoms and severity levels [12]. Both studies included children with dysentery; however, MAL-ED included children who had at least one stool containing blood, while GEMS did not count cases that had fewer than three stools, irrespective of blood [9], [12]. The different inclusion criteria and disease severity between the two studies is important to consider when reviewing their conclusions.

### 4.3 Diagnosis

Diagnostic techniques vary in their ability to detect infectious agents, therefore affecting attribution of diarrheal etiology. Ideally, diagnostic tests for diarrheal illness in LMICs would be quantitative, reliable, sensitive, specific, rapid, and accessible [56]. Currently, no diagnostic methods for ETEC and *Shigella* adhere to all these criteria.

*Shigella* spp. are conventionally diagnosed by isolation on culture, subsequent standard biochemical testing, and serotyping via serum agglutination [57], [29], [58]. ETEC infection is generally diagnosed by selecting suspected colonies from differential media and using PCR to detect the presence of ETEC-specific genes, such as those encoding LT or ST, or by using an ELISA [57], [58], [59]. These methods tend to be more standardized and less dependent on expensive equipment [57], [58], [60], [61].

Conventional approaches, particularly involving cultures, can be less sensitive than molecular techniques [61]. Isolating colonies of particular pathogens from stool can be time consuming and difficult, especially if antibiotics were used or if pathogens, including *Shigella*, are fastidious [26], [60]**.** Both *E. coli* and *Shigella* spp. can also remain in non-dividing states when cultured [62], [63]. Assay accuracy and sensitivity can vary with bacterial load, or the number of colonies selected [58], [64]. The significant constraints and challenges of these conventional methods have driven the use of newer PCR assays for pathogen identification.

Taqman Array Cards (TAC), and Loop-mediated Isothermal Amplification (LAMP) are two examples of next-generation assays that are being increasingly used for enteric diagnostics [65], [66]. The TAC qPCR platform has been used to reanalyze GEMS and MAL-ED samples [47], [48]. TAC involves arrayed singleplex reactions that can run many small volume qPCRs against different pathogens simultaneously but separately, enabling a larger array of pathogens to be tested [65], [67]. These assays are also now increasingly being utilized in disease surveillance. LAMP assays are isothermal nucleic acid amplification tests that have been preliminarily tested in a quantitative or semi-quantitative function and are highly specific due to the use of several primers [66], [68], [69].

These newer molecular assays are generally much more sensitive than conventional methods [70]. qPCR assays, such as TAC, have an expanded range of genes to identify more specifically ETEC colonization factors and *Shigella* serotypes, and can also be used to quantify the amount of pathogen present, based on the number of PCR amplification cycles (Cq) [60], [70]. Thus, quantitative methods can indicate the etiologic agent by detecting the most common pathogen among several co-infecting microbes [60]. TAC and LAMP are also generally less time consuming than conventional culture, with LAMP complete in under one hour [69], [71].

However, there are various challenges in selecting the optimal detection methodology. Assay sensitivity can vary by pathogen type, leading to possible bias when assessing particular pathogens [26], [67], [72]. Additionally, stool has inhibitory components that can interfere with DNA extraction and amplification [60], [67], [70]. Finally, molecular assays tend to rely on materials that are impractical to use in LMICs [66]. LAMP may eventually become an accessible LMIC diagnostic, while regular use of qPCR may only be possible in research settings or regional surveillance laboratories.

Importantly, laboratory procedures including primers, reagents, and protocols need to be standardized across different laboratory settings to ensure comparable data [67], [73]. Studies assessing these molecular techniques have acknowledged that variation across protocols leads to differing results [74]. For example, MAL-ED data indicated that a one-unit difference in Cq cut-off led to at least a 3% change in attributable fraction for *Shigella* [47]. The high sensitivity of TAC may increase the likelihood of detecting several co-infecting organisms; thus, determining the Cq cut-off value that is used to assign a specific disease etiology for each pathogen is important [47].

### 4.4 Co-infection, asymptomatic infection, and infections of unknown etiology

Establishing the etiology of diarrhea is complex due to the common presence of co-infections and asymptomatic carriage of pathogens [48], [60], [75]. The MCEE/CHERG stratified analyses by single as well as multi pathogen approaches because prior research suggests that single pathogen studies tend to result in higher estimates, though there is debate over the extent of these differences [10], [76]. GBD estimates did include single pathogen studies but reported using discretion in assessing potential biases [10], [43], [76]. In multiple studies, almost half of the diarrheal cases had no assignable cause, often due to a lack of information or to a possible unassessed etiologic agent [14], [39].

GEMS found that 72% of asymptomatic controls carried at least one diarrheal pathogen, indicating that detecting the presence of a pathogen may not be clinically relevant [9]. Regression methods were used to find which pathogens were significantly associated with diarrhea in cases, compared to controls, and the AF was calculated based on these analyses [9]. *Shigella* had a high AF because about 90% of positive cases had diarrhea. Children infected with ETEC producing ST exhibited higher rates of asymptomatic carriage, so only about 60–70% of those cases could be attributed to that pathogen [9]. While original GEMS analyses involved established *Shigella* or ETEC etiology using culture or multiplex PCR, respectively, the qPCR reanalysis provided a more quantifiable approach [48].

## 5 The long-term health consequences of diarrheal infections

While the estimates of disease burden previously discussed primarily focused on mortality or diarrheal incidence, there are increased efforts to quantify the long-term economic and health-related effects of enteropathogens in order to better understand the relationship between diarrhea and the perpetuation of poverty.

According to the World Bank, 1 in 10 people globally live in extreme poverty, and over half of this population consists of young, poorly educated communities in rural Sub-Saharan Africa [77]. A lack of safe water and adequate sanitation facilitate high rates of enteric infection, which can cause blunting of gut villi that impede nutrient absorption leading to malnutrition, and indirect effects such as growth stunting and inadequate immune responses and to immunization [4], [78]. Furthermore, data reviewed by Guerrant et al. suggests that stunted growth in early childhood is a risk factor for cognitive impairment, as well as other co-morbidities such as obesity, type 2 diabetes, metabolic syndrome and cardio vascular disease (CVD) later in life [4]. A recent publication suggests that diarrhea is also associated with acute lower respiratory tract infection in young children, in LMICs [79]. *Shigella* and ETEC, in particular, have been significantly associated with reduced childhood growth compared to other common pathogens such as rotavirus [41], [59], [80].

A challenge with evaluating the impact of a vaccine on indirect, long term sequelae of diarrhea is that measuring such effects can require costly longitudinal studies that are often not practical for vaccine trials [36], [80], [81], [82]. While stunting is potentially associated with a broad spectrum of morbidities, there are no validated biomarkers or clinical diagnostic criteria to evaluate the causative mechanisms [83]. It is therefore difficult to stratify the effects of diarrhea, malnutrition, and stunting on long term health and economic development. It is important to consider how to capture the impact of these long-term to measure the entire burden of these diseases, so that the full benefit that a vaccine may offer may be measured. Further evaluation of the pathologic mechanisms by which ETEC- and *Shigella*-associated diarrhea impact growth, cognition, and future disease incidence may validate a proxy or correlate that will facilitate this.

## 6 The potential public health impact of a *Shigella* and ETEC vaccine

The elements that influence the decision to introduce a vaccine are based on (i) the public health need for the vaccine, i.e. its potential to reduce both mortality and morbidity, in the context of other available interventions, (ii) the characteristics of the vaccine, such as its safety and efficacy, as well as its cost-effectiveness and (iii) the capacity of the immunization program and the underlying health system to deliver the vaccine [84]. These factors determine the evidence, or data, that will be required for a policy review if these vaccines become licensed.

In addition to decreasing the mortality burden, a vaccine would significantly reduce the large costs of treating diarrhea, and the loss of productivity of adults when they or their children are ill [34], [85]. The rise in antibiotic resistance, particularly for the treatment of *Shigella*, will likely increase the cost and complexity of treatment regimens that would be obviated if an effective vaccine were available. An important next step will be to further characterize and substantiate the potential health and socio-economic benefits that vaccination could provide in order to better articulate the potential vaccine impact, and strengthen the value proposition for developing these vaccines [86]. To date, there have been studies describing the economic value of ETEC and *Shigella* vaccines among travelers and military from high income countries; however, these study populations may not capture the effects of vaccination for children in endemic countries [87]. An investment market assessment on a vaccine for ETEC stand-alone vaccines suggested it may present an estimated annual revenue potential of more than $600 million, 10  years after global launch, based on primarily on travelers and middle-income markets (both public and private), but military and low-income markets were also represented [88]. Finally, it will be important to understand the timeline and probability of technical success for the various vaccine approaches, and to model both the potential burden estimate and need for these vaccines when programmatically suitable ETEC and *Shigella* vaccine combinations may become available.

## 7 Concluding remarks and recommended discussion points

The studies reviewed here substantiate that both *Shigella* and ETEC cause acute and long-term health and economic consequences. However, the point estimates of mortality differ between iterations published by the same group, as well as between estimates of different groups, although the uncertainty intervals are broad and overlapping. It is unclear how much of the fluctuation in estimates is a result of modifications to detection and modelling methodology versus true changes in the number of deaths caused by disease, and there is no consensus as to which estimates are the most accurate or consistent going forward. In the interim, these estimates are informing and impacting the prioritization of vaccine candidates, and may result in the early termination of the development of a potentially effective vaccine. Better certainty is needed to support decision making for investment decisions, and ultimately vaccine introduction.

Both the GBD and MCEE estimates are currently undergoing revision, and are expected to be published in late 2017. Since several differences in the methodology of these two models remain, they are unlikely to converge. For this reason, we believe that broader discussion and investigation into the underlying reasons for the uncertainties and ranges in mortality estimates are warranted to help prioritize public health goals for these vaccines, and ultimately guide policy decision making and vaccine introduction. We also would like to understand how the indirect morbidity effects of diarrhea caused by these pathogens could be measured, and how these could contribute to the cost-effectiveness assessment of potential vaccines.

The following aspects are recommended as priority areas of focus:

• Understanding the uncertainties and limitations in the mortality modelling methodologies that are currently available, including the role that sensitive TAC analyses have played in shaping diarrheal morbidity and mortality estimates;
• An improved understanding of the proportional distribution of inpatient vs outpatient cases of *Shigella* and ETEC is needed to better inform burden assessments and to rationalize the most cost-effective vaccination strategy;
• Assessment of long-term morbidity and socio-economic impact of ETEC and *Shigella* diarrhea and potential impact of vaccination in low-income pediatric populations;
• The difference in timeline and development cost; risk (including probability of technical success) and eventual uptake of ETEC and *Shigella* vaccine candidates; and the mortality and morbidity burden within this intervening period to facilitate prioritization;
• Assessment of global ETEC and *Shigella* surveillance to improve the epidemiological understanding of ETEC and *Shigella* diarrheal disease burden, particularly considering potential geographical heterogeneity and antigenic variation of circulating strains;
• An important next step will be to further characterize and substantiate the potential health and socio-economic benefits that vaccination could provide in order to better articulate the potential vaccine impact, and strengthen the value proposition for developing these vaccines.

Convergence on these issues is critical as the current ETEC and *Shigella* vaccines advance to late stage clinical development, to inform effective decision making for future enteric vaccine development. As an initial next step, WHO convened a global consultation to better understand the status and challenges of determining of ETEC and *Shigella* burden of disease estimates, and the strategic direction of vaccine development against these antigens.
